# Characteristics of Acute kidney injury in Xizang: a retrospective analysis from the largest tertiary hospital in the Xizang Autonomous region, China

**DOI:** 10.1007/s40620-025-02395-2

**Published:** 2025-09-11

**Authors:** lang-jie Chi-lie, Ya-Hui Yang, Lei Zhang, Wen Tang, Yong A

**Affiliations:** 1 Department of Nephrology, People’s Hospital of Xizang Automomous Region, No.18, North Linkuo Road, Chengguan District, Lhasa, 850000 Xizang China; 2 Department of Nephrology, Peking University Third Hospital, Beijing, 100191 China

**Keywords:** Acute kidney injury, Xizang, Mortality

Acute kidney injury (AKI) imposes a substantial global burden of morbidity and mortality [1–3]. A recent nationwide survey in China revealed an all-cause mortality rate of 12.4% among AKI patients [4]. However, the Xizang Autonomous Region of China was not included in this analysis, despite the growing body of literature documenting AKI characteristics and outcomes across Chinese regions [4]. Consequently, the clinical phenotypes, prognostic determinants, and healthcare outcomes of AKI in this region  remain largely unexplored [5–7]. Given the Xizang Autonomous Region of China’s high-altitude environment (mean elevation > 4000 m), coupled with distinct geographical, climatic, socioeconomic, and cultural factors, there is an urgent need to characterize AKI epidemiology and management patterns in this region. This single-center, retrospective cohort study was therefore conducted at the largest tertiary hospital in the region: the People’s Hospital of Xizang Autonomous Region of China. Established as one of the four tertiary care facilities in Lhasa, the hospital currently has 901 inpatient beds and serves as the definitive referral center for critically ill patients across the Xizang region.

Clinical data were retrospectively extracted from the electronic health records (EHRs) of patients admitted between January 1, 2014, and August 14, 2023. Patients were included if they had a diagnosis of AKI, as coded by ICD-10-CM, that was subsequently confirmed by nephrologists through a review of medical records, following the 2012 Kidney Disease: Improving Global Outcomes (KDIGO) definition of AKI. Duplicate cases were excluded. The study protocol was approved by the Ethics Committee of the People's Hospital of Xizang Autonomous Region of China (Approval No. ME-TBHP-24-kJ-001). Given the retrospective design of the study, informed consent was waived.

The following data were collected: demographic information, altitude, incidence of altitude sickness, comorbidities, nephrotoxic medications, invasive procedures, critical illnesses, AKI classification, AKI staging, kidney replacement therapy, and referral to a nephrologist. All-cause in-hospital mortality data were also gathered.

Statistical method is described in Supplementary material.

In the setting and period of study a total of 691 patients were diagnosed with acute kidney injury (AKI) among 186,170 hospitalizations. The overall percentage of diagnosis of AKI was 0.37% (0.10% ,0.16%, 0.25%, 0.49%, 0.38%, 0.36%, 0.48%, 0.54% and 0.40%, from 2014 to 2022, respectively).

The majority of AKI cases were recorded in medical departments (66.14%), followed by the intensive care unit (ICU) (22.14%), surgical departments (8.97%), and other departments (2.7%). Age of AKI patients was 53.85 ± 18.59 years, with a male predominance (67.1%). Five hundred-five (73.1%) patients presented with severe critical illness, and 165 (23.9%) with AKI stage 3. Pre-renal AKI accounted for more than half of the cases (60.1%), with renal hypoperfusion (49.5%) and other critical illnesses (34.7%) being the most common causes. Among the factors contributing to renal hypoperfusion, hypovolemia (35.2%) and decreased cardiac output (18.2%) were the most frequent ones. A higher proportion of pre-renal AKI cases was observed in non-nephrology departments compared to nephrology departments (78.0% vs. 21.1%; *p* < 0.001). Non-nephrology departments were more likely to report renal hypoperfusion as the causative factor (64.9% vs. 16.1%;* p* < 0.001). Furthermore, acute cerebrovascular disease (19.5% vs. 1.8%; *p* < 0.001), acute cardiovascular disease (15.0% vs. 4.6%; *p* < 0.001), and acute respiratory failure (15.2% vs. 4.1%;* p* < 0.001) were more common in non-nephrology departments compared to nephrology departments. In nephrology departments, a greater proportion of AKI patients had pre-existing chronic kidney disease (CKD) (16.1% vs. 3.4%; *p* < 0.001) and required kidney replacement therapy (28.9% vs. 8.7%; *p* < 0.001). Additionally, kidney biopsy was more frequently performed in nephrology departments (11.9% vs. 1.3%; *p* < 0.001).

Overall, 64.1% of AKI patients were referred to a nephrologist. Rates of referral were 55.3%, 68.4%, and 83.0% for patients with AKI stages 1, 2, and 3, respectively. Only 47.6% of AKI patients from non-neprhology departments were referred to a nephrologist, and surprisingly, 77.8% of AKI patients from the ICU were not referred.

The overall in-hospital mortality rate for AKI patients was 12.4% (86 out of 691 cases). Among the 86 in-hospital deaths, the most common cause of death was multiple organ failure, accounting for 47 (54.7%) deaths. Notably, patients who received a referral to a nephrologist had a significantly lower mortality rate (9.3%) compared to those who did not (18.1%, *p* < 0.001). Acute respiratory failure, surgical operations and AKI stages 2 or 3 (with stage 1 as the reference) were associated with higher in-hospital mortality. In contrast, referral to a nephrologist was found to be independently associated with a lower in-hospital mortality rate (Table 1).

**Table 1:** Logistic regression analyses for factors associated with all-cause in-hospital mortality of AKI patients

Variable	Univariate analysis		Multivariable analysis	
	OR (95% CI)	*p* value	OR (95%CI)	*p* value
Polycythemia	1.820 (0.772, 4.319)	0.170	–	–
Altitude sickness	1.621 (0.727, 3.614)	0.238	–	–
Infection	1.172 (0.735, 1.869)	0.504	–	–
Sepsis	0.776 (0.177, 3.406)	0.737	–	–
Multiple organ failure	4.227 (2.683, 6.816)	< 0.001	NA	–
Tumor	1.608 (0.752, 3.437)	0.221	–	–
Acute cardiovascular disease	0.990 (0.489, 2.001)	0.977	–	–
Acute cerebrovascular disease	1.788 (1.010, 3.167)	0.046	NA	–
Acute respiratory failure	7.225 (4.273, 12.216)	< 0.001	6.808 (3.927, 11.805)	< 0.001
Acute liver failure	3.317 (1.762, 6.245)	< 0.001	2.612 (1.303, 5.234)	0.007
Ileus/intestinal necrosis	1.005 (0.224, 4.501)	0.995	–	–
Urolithiasis	0.497 (0.064, 3.825)	0.502	–	–
Kidney failure caused by parenchymal disease	0.423 (0.179, 0.998)	0.050	NA	–
DKA/HHS	1.422 (0.403, 5.016)	0.584	–	–
Surgery	2.238 (1.252, 3.999)	0.007	2.114 (1.131, 4.064)	0.019
Combined obstetric conditions	1.005 (0.122, 8.270)	0.996	–	–
Chronic Kidney Disease	0.750 (0.290, 1.943)	0.554	–	–
Pulmonary heart disease	0.327 (0.043, 2.464)	0.278	–	–
Diabetes mellitus	1.406 (0.740, 2.672)	0.298	–	–
Hepatitis C	< 0.001	0.999	–	–
Acute pancreatitis	1.829 (0.837, 3.998)	0.113	–	–
Other hematological neoplasms	1.768 (0.195, 16.002)	0.612	–	–
Kidney Biopsy	0.218 (0.029, 1.617)	0.136	–	–
AKI stage				
1 (Reference)	Reference	0.096	Reference	0.047
2	1.862 (1.055, 3.286)	0.032	2.237 (1.198, 4.177)	0.012
3	1.309 (0.756, 2.267)	0.336	1.814 (0.974, 3.378)	0.061
Referral to a nephrologist (yes)	0.460 (0.292, 0.726)	0.001	0.506 (0.301, 0.851)	0.010
Constant	–	–	0.083	< 0.001

Significant variables identified by univariate binary logistic regression (*p* < 0.1) were subsequently included in multivariate logistic regression model to assess their impact on in-hospital mortality. Variable(s) entered to multivariable analysis: multiple organ failure, acute cerebrovascular disease, acute respiratory failure, acute liver failure, kidney failure caused by parenchymal disease, surgery, hypertension, AKI stage (AKI stage 1 as the reference category) and referral to a nephrologist

Final variables associated with in-hospital mortality were assessed by using the forward stepwise (ward) method in logistical regression, with a *p*-value of < 0.05 considered statistically significant

*AKI* acute kidney injury, *DKA* diabetic ketoacidosis, *HHS* hyperglycemic hyperosmolar status

This study, based on hospitalization data from the largest tertiary hospital in the XizangAutonomous Region of China, is the first to investigate the characteristics and factors associated with the outcomes of AKI in this region. Our findings provide important insights and offer preliminary data for future research focused on AKI prevention, treatment, and improved management in this region.

Our study revealed that the highest number of AKI cases (66.14%) occurred in medical departments and 22.14% of AKI cases were treated in the ICU. The proportion of AKI among ICU patients in our study aligns with the findings of Yang et al. [4], but differs from, and is lower than, the rates commonly reported in the literature. The possible explanation is that the proportion of ICU beds in the studied hospital is substantially lower than that of medical department. Besides, as a local hospital, community-acquired AKI is the main form of AKI and these patients are mainly admitted to medical departments.

However, this study only included patients diagnosed with AKI based on the diagnosis recorded in hospitalization charts and potential selection bias exists. Second, the retrospective design and single-center study design limit the generalizability of our findings. Third, although factors associated with outcome were investigated, causality cannot be inferred. Therefore, further multi-center prospective studies are needed to validate our results. The overall AKI diagnosis rate is lower compared to previous studies but very close to the reported rates from China of overall AKI detected in Southwestern or Northwestern Chinese local hospitals (0.19%–0.64%) [4]. The lower diagnostic rate may reflect lower awareness of AKI. Despite these limitations, the strengths of the study include its thorough evaluation, as well as being the first comprehensive report on AKI in the Xizang Autonomous Region, China.

## Data Availability

The datasets used and analyzed during the current study are available from the corresponding author on reasonable request.

## Abbreviations

AKI: Acute kidney injury
ICUs: Intensive care units
CKD: Chronic kidney disease
KDIGO: Kidney Disease: Improving Global Outcomes
OR: Odds ratio
CI: Confidence interval
SD: Standard deviation
